# Heat source and sink effects on periodic mixed convection flow along the electrically conducting cone inserted in porous medium

**DOI:** 10.1371/journal.pone.0260845

**Published:** 2021-12-23

**Authors:** Asifa Ilya, Muhammad Ashraf, Aamir Ali, Zahir Shah, Poom Kumam, Phatiphat Thounthong

**Affiliations:** 1 Department of Mathematics, Faculty of Science, University of Sargodha, Sargodha, Pakistan; 2 Department of Mathematics, COMSATS University Islamabad, Attock, Pakistan; 3 Department of Mathematical Sciences, University of Lakki Marwat, Lakki Marwat, Khyber Pakhtunkhwa, Pakistan; 4 Fixed Point Research Laboratory, Fixed Point Theory and Applications Research Group, Center of Excellence in Theoretical and Computational Science (TaCS-CoE), Faculty of Science, King Mongkut’s University of Technology Thonburi (KMUTT), Thung Khru, Bangkok, Thailand; 5 Department of Medical Research, China Medical University Hospital, China Medical University, Taichung, Taiwan; 6 Department of Teacher Training in Electrical Engineering, Renewable Energy Research Centre, Faculty of Technical Education, King Mongkut’s University of Technology North Bangkok, Bangsue, Bangkok, Thailand; Central University of Karnataka, INDIA

## Abstract

The system of partial differential equations governing the unsteady hydromagnetic boundary-layer flow along an electrically conducting cone embedded in porous medium in the presence of thermal buoyancy, magnetic field, heat source and sink effects are formulated. These equations are solved numerically by using an implicit Finite-Difference Method. The effects of the various parameters that are source/sink parameter, porous medium parameter, Prandtl number, mixed convection parameter and magnetic Prandtl number on the velocity, temperature profiles, transverse magnetic field are predicted. The effects of heat source and sink parameter on the time-mean value as well as on transient skin friction; heat transfer and current density rate are delineated especially in each plot. The extensive results reveal the existence of periodicity and show that periodicity becomes more distinctive for source and sink in the case of the electrically conducting cone. As the source and sink contrast increases, the periodic convective motion is invigorated to the amplitude and phase angle as reflect in the each plot. The dimensionless forms of the set of partial differential equations is transform into primitive form by using primitive variable formulation and then are solved numerically by using Finite Difference Scheme which has given in literature frequently. Physical interpretations of the overall flow and heat transfer along with current density are highlighted with detail in results and discussion section. The main novelty of the obtained numerical results is that first we retain numerical results for steady part and then used in unsteady part to obtain transient skin friction, rate of heat transfer and current density. The intensity of velocity profile is increased for increasing values of porosity parameter Ω, the temperature and mass concentration intensities are reduced due heat source effects.

## 1. Introduction

Several studies have been conducted on heat transport through a porous medium due to its industrial applications and numerous technical processes. Flows through porous medium are of particular interest because they are so common in nature and daily life. Water is saturated in porous materials such as sand and underground crushed rocks, which allows the fluid to move and be transported through the material under the influence of local pressure gradients. This fascination stems from the various practical applications that can be modeled or approximated, through porous medium such as packed sphere beds, high-performance building insulation, grain storage, chemical catalytic reactors, sensible heat storage beds and heat exchange between soil and atmosphere. Moreover, soil salt leaching, solar power collectors, electrochemical processes, filtering devices, insulation of nuclear reactors, regenerative heat exchangers, geothermal energy systems and many other areas. Convective heat transfer in porous medium is of much interest of research community engaged in different applied and engineering disciplines.

Kamel [1] proposed the problem of one-dimensional MHD incompressible viscous fluid flow due to heat and mass transfer through a porous medium bounded by an infinite vertical porous plate. Chamkha *et al*. [2] investigated the steady-state, hydromagnetic two dimensional forced convective boundary-layer flow of incompressible Newtonian, electrically-conducting, and heat-generating/absorbing fluid over a non-isothermal wedge with permeable surface in the existence of thermal radiation effects. He observed that as the wall mass transfer parameter is increased, both the local skin-friction coefficient and the local Nusselt number are increased. The effects of internal heat generation, suction, and injection on heat transfer in a porous medium over a stretching surface have been investigated by Elbashbeshy and Bazid [3]. Cortell [4] encountered the influence of two dimensional fluid flow and heat transfer in porous medium over a stretching surface. The flow is affected by stretching the surface linearly with internal heat generation or absorption, as well as the presence of suction, blowing, and surface impermeability. He found that the Prantdl number has a tendency to lower the temperature as it rises. In the presence of a magnetic field, the issue of heat and mass transfer on a stretching sheet in a visco-elastic fluid flow through a porous medium with heat generation or absorption has been numerically investigated by Seddeek [5].

Sharma and Singh [6] predicted the problem of Variable thermal conductivity and heat source/sink impact on flow of a viscous incompressible electrically conducting fluid near a stagnation point on a non-conducting stretching layer in the presence of a uniform transverse magnetic field and variable free stream velocity. Later in the presence of a transverse magnetic field and a heat source, the effect of mass transfer on free convective flow and heat transfer of a viscous incompressible electrically conducting fluid past a vertical porous plate through a porous medium with time-dependent permeability highlighted by Das *et al*. [7]. Pal and Mondal [8]. Analyzed the effects of temperature-dependent viscosity on non-Darcy MHD mixed convective boundary layer flow and heat transfer past a porous medium in the presence of non-uniform heat source/sink. They found that the effect of non-uniform surfaces and temperature-dependent heat source/sink parameters is to produce temperature for heat source and absorb temperature for heat sink values. As a result, the cooling performance of a non-uniform heat sink is dominant. Abel *et al*. [9] conducted the analysis on the effects of laminar, two dimensional flow of an incompressible second-grade non-Newtonian liquid due to a stretching sheet through a porous medium under the influence of an external magnetic field. In the presence of a heat source or sink, the effects of thermal radiation and heat transfer over an unsteady stretching surface embedded in a porous medium have been discussed by Elbashbeshy, *et al*. [10]. Osman *et al*. [11] computed the effects of thermal radiation and chemical reactions on unsteady MHD convective flow of an incompressible fluid through a porous medium confined by an infinite vertical plate. Zheng *et al*. [12] discussed the behavior of heat transfer and boundary layer flow on time dependent permeable stretching sheet with a non-uniform heat source/sink. Later in the presence of a heat source or sink, the effects of heat transfer and thermal radiation over an unsteady stretching surface fixed in a porous medium are studied by Elbashbeshy and Emam [13].

The combined effects of a magnetic field and convective diffusion of species through a non-Darcy porous medium over a vertical non-linear stretching sheet in the existence of Ohmic dissipation and a non-uniform heat source/sink have been depicted by Pal and Mondal [14]. For transpiration cases, the effects of thermophoretic and heat source/sink parameters on MHD flow over an inclined radiate isothermal permeable surface have been explored by Noor *et al*. [15]. The combined effects of transverse magnetic field and heat source on free convective flow of viscoelastic fluid along permeable plate immersed in porous medium have been explored by Mishra *et al*. [16]. The magnetohydrodynamic convective boundary layer flow in the presence of heat generation and viscous dissipation effects past a stretching sheet inserted in porous medium carried out by Dessie and Kishan [17]. Pal and Mandal [18] discussed the effects of viscous dissipation and thermal radiation on steady two-dimensional mixed convection boundary layer flow towards a stagnation-point flow over a stretching/shrinking sheet fixed in porous medium. They found that While a sheet is stretched, the suction parameter reduces the velocity and temperature profiles, while when a sheet is shrunk, the opposite trend is observed.

Chaudhary *et al*. [19] briefly described that a boundary layer analysis is used to examine the effects of thermal radiation on the flow of an incompressible viscous electrically conducting fluid over an unsteady stretching sheet embedded in a porous medium in the presence of a heat source or drain. Sandeep and Sulochana [20] examined the influence of heat transfer of non-uniform source/sink on micropolar fluid over a stretching/shrinking sheet. The existence of a heat source/sink, MHD flow and heat transfer of a couple stress fluid over an oscillatory stretching sheet embedded in a porous medium have been illustrated by Ali *et al*. [21]. They noted that by increasing the heat source parameter, the temperature rises. In the presence of non-uniform heat source and first order chemical reaction the phenomenon of heat has been carried out to stretching sheet immersed in porous medium by Tripathy*et al*. [22]. Hayat *et al*. [23] investigated thatthe Internal heat generation and absorption have an effect on a nonlinear boundary layer flow of an upper-convected Maxwell (UCM) fluid over a permeable wall. The steady boundary layer magnetohydrodynamic stagnation-point flow past a stretching sheet through porous media in the presence of a heat source/sink. has been studied by Mishra *et al*. [24]. Suneetha *et al*. [25] gave an attention to the study of steady two dimensional buoyancy effects on MHD flow through a porous medium over a permeable stretching layer in the presence of suction/injection. Kumar *et al*. [26] examined the hydromagnetic three-dimensional flow of a radiating Maxwell fluid over a stretching sheet embedded in a porous medium with Soret effect and heat source/sink, first-order chemical reaction. Vijayalakshm *et al*. [27] investigate the effects of electro magneto hydrodynamics on the fluid transportation properties of a chemically reacting Casson fluid with two different geometries. The physical behavior of the combined effect of nano particle material motion and heat generation/absorption due to the effect of different parameters involved in the prescribed flow model have been studied by Ashraf *et al*. [28]. By exerting a magnetic field exact at the surface of the magnetized cone, periodic mixed convection flow and heat transfer characteristics can be achieved by Ilyas *et al*. [29]. Unsteady, two dimensional boundary–layer fluid flow mechanism across a nonconducting horizontal circular cylinder immersed in porous medium has been carried out by Ashraf *et al*. [30]. Later, Kumar *et al*. [31–37] discussed the Newtonian and non-Newtonian magnetohydrodynamics convective heat transfer mechanism for different characteristics of heat and fluid flow around different geometries. Dynamics of unsteady MHD flow with thermophoresis of particles and variable thermo physical properties along different shapes has been studied by Animasaum and his co-authors [38–41].

In keeping view the above literature, we interact with the phenomenon for unsteady mixed convective flow across the surface of electrically conducting cone embedded in porous medium in the presence of heat source and sink. The time dependent dimensionless equations which illustrate the hydromagnetic laminar flow along the surface of sphere are formulated. Further, we consider the motion along sphere within plume region-I with main stream velocity. By using Stokes condition we separate steady and unsteady part from the modeled partial differential equation. Later, the unsteady part is further splited in to real and imaginary part. First we secure numerical solutions for steady part and then used in unsteady part to calculate periodic skin friction, heat transfer, and current density along the surface of the cone embedded in porous medium. We also calculate fluid velocity, magnetic field and temperature profiles, to ensure the correctness of numerical results by satisfying the prescribed boundary conditions.

## 2. Formulation and coordinate system

**Fig 1:**
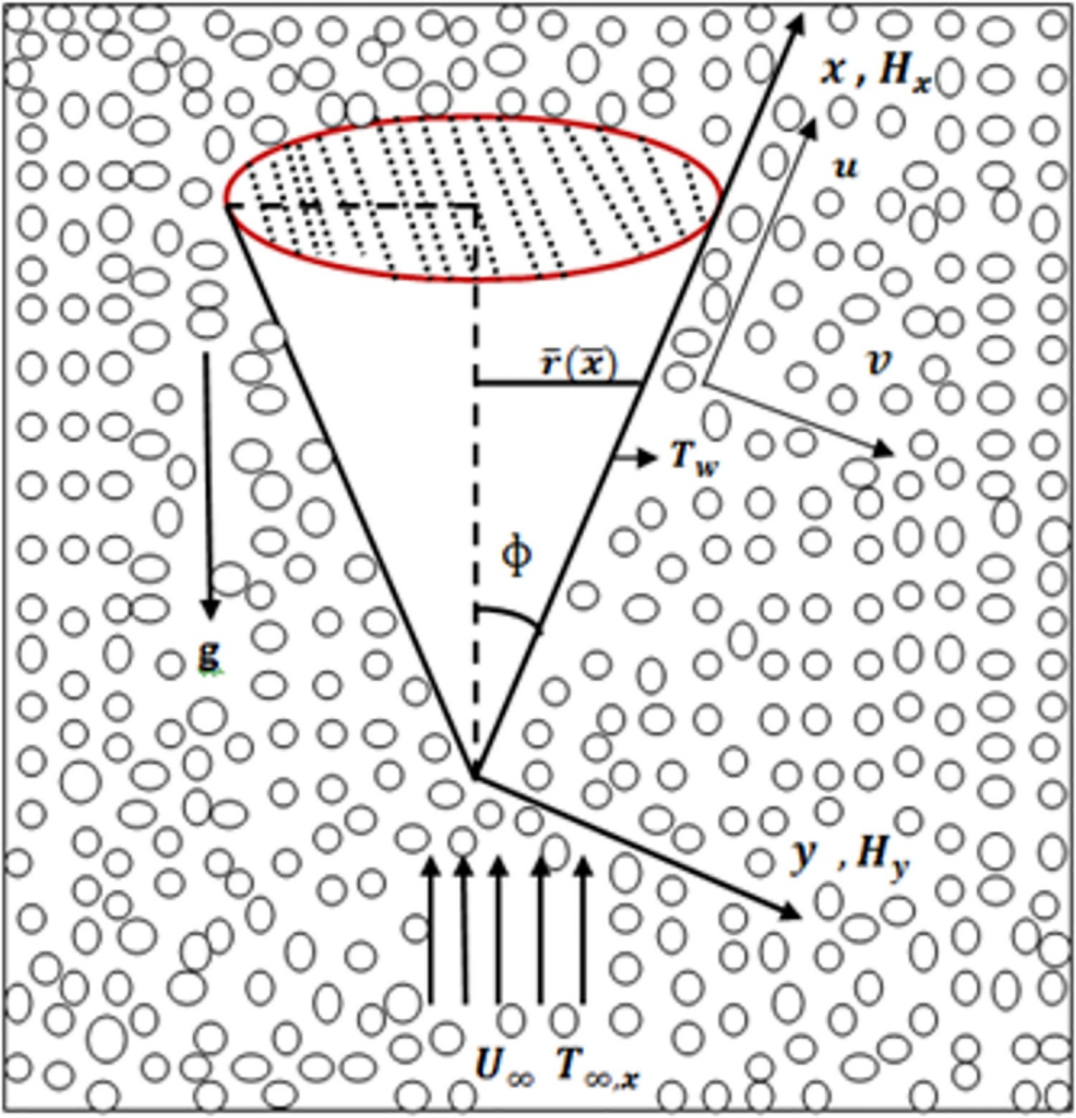
Electrically conducting cone and coordinate system.

Consider a two-dimensional periodic mixed convection boundary-layer fluid flow along the surface of thermally and electrically conducting cone embedded in porous medium. The scheme of coordinates is shown in Fig 1. Coordinate *x* is measured along the surface and *y* is measured normally on the cone surface. The velocities *u* and *v* along the (*x*, *y*)-direction, *H*_*x*_ represent the component of magnetic field at the surface of cone, *H*_*y*_ component is taking normal to the surface of cone and external fluid velocity of the cone is *U*(*x*, *t*). Moreover, magnetic field intensity proceeds exact at the surface of the cone. The form of governing dimensioned continuity, momentum, magnetic, and energy equations, as well as the boundary conditions are given as below

$$\frac{\partial u}{\partial x}+\frac{\partial v}{\partial y}=0$$ (1)

$$\epsilon^{+}\left(\frac{\partial u}{\partial t}+u\frac{\partial u}{\partial x}+v\frac{\partial u}{\partial y}\right)=\epsilon^{+}\nu \frac{\partial^{2}u}{\partial y^{2}}+\epsilon^{+}\frac{\mu }{\rho }\left(H_{x}\frac{\partial H_{x}}{\partial x}+H_{y}\frac{\partial H_{x}}{\partial y}\right)-{\epsilon^{+}}^{2}\frac{\mu }{k}(u)+\epsilon^{+}g\beta (T-T_{\infty })\cos(\frac{x}{a})$$ (2)

$$\frac{\partial H_{X}}{\partial x}+\frac{\partial H_{y}}{\partial y}=0$$ (3)

$$\frac{\partial H_{x}}{\partial t}+u\frac{\partial H_{x}}{\partial x}+v\frac{\partial H_{x}}{\partial Y}-H_{x}\frac{\partial u}{\partial x}-H_{y}\frac{\partial u}{\partial y}=\gamma_{m}\frac{\partial^{2}H_{x}}{\partial y^{2}}$$ (4)

$$\frac{\partial \theta }{\partial t}+u\frac{\partial \theta }{\partial x}+v\frac{\partial \theta }{\partial y}=\alpha \frac{\partial^{2}\theta }{\partial y^{2}}\pm \frac{Q_{o}}{\rho C_{p}}(T-T_{\infty }).$$ (5)

The dimensioned form of boundary conditions are

$$\begin{array}{c} u=v=0,\;H_{y}=0,\;H_{x}=H_{0},\;T=T_{W},\;at\;y=0\\ u\to U(\tau ),\;H_{x}\to 0,\;T\to T_{\infty }\;as\;y\to \infty . \end{array}$$ (6)

The following are the dimensionless system of coupled nonlinear partial differential equations:

$$\frac{\partial \bar{u}}{\partial \bar{x}}+\frac{\partial \bar{v}}{\partial \bar{y}}=0$$ (7)

$$\frac{\partial \bar{u}}{\partial \tau }+\bar{u}\frac{\partial \bar{u}}{\partial \bar{x}}+\bar{v}\frac{\partial \bar{u}}{\partial \bar{y}}=\frac{\partial^{2}\bar{u}}{\partial {\bar{y}}^{2}}+\xi \left({\bar{h}}_{x}\frac{\partial {\bar{h}}_{x}}{\partial \bar{y}}+{\bar{h}}_{y}\frac{\partial {\bar{h}}_{x}}{\partial \bar{y}}\right)-\Omega (\bar{u})+\lambda \bar{\theta }$$ (8)

$$\frac{\partial {\bar{h}}_{x}}{\partial \bar{x}}+\frac{\partial {\bar{h}}_{y}}{\partial \bar{y}}=0$$ (9)

$$\frac{\partial {\bar{h}}_{x}}{\partial \tau }+\bar{u}\frac{\partial {\bar{h}}_{x}}{\partial \bar{x}}+\bar{v}\frac{\partial {\bar{h}}_{x}}{\partial \bar{y}}-{\bar{h}}_{x}\frac{\partial \bar{u}}{\partial \bar{x}}-{\bar{h}}_{y}\frac{\partial \bar{u}}{\partial \bar{y}}=\frac{1}{\gamma }\frac{\partial^{2}{\bar{h}}_{x}}{\partial {\bar{y}}^{2}}$$ (10)

$$\frac{\partial \bar{\theta }}{\partial \tau }+\bar{u}\frac{\partial \bar{\theta }}{\partial \bar{x}}+\bar{v}\frac{\partial \bar{\theta }}{\partial \bar{y}}=\frac{1}{P_{r}}\frac{\partial^{2}\bar{\theta }}{\partial {\bar{y}}^{2}}\pm \delta \theta .$$ (11)

The dimensionless boundary conditions are:

$$\begin{array}{c} \bar{u}=\bar{v}=0,\;{\bar{h}}_{y}=0,\;{\bar{h}}_{x}=1,\;\bar{\theta }=1,\;at\;\bar{y}=0\\ \bar{u}\to \bar{U}(\tau ),\;\bar{\theta }\to 0,\;{\bar{h}}_{x}\to 0,\;as\;\bar{y}\to \infty . \end{array}$$ (12)

The third and fourth term on right hand side of Eq (8) which are Ω and *λ*, represents porosity number and mixed convection dimensionless number, γ is Prandtl magnetic number, Prandtl parameter is Pr, *H*_*o*_ is the exact strength of the magnetic field at the surface and *δ* represents the heat generation absorption parameter.


$$\xi =\frac{\mu {H_{0}}^{2}}{\rho {U_{\infty }}^{2}},\;\alpha =\frac{\kappa }{\rho C_{p}},\;\lambda =\frac{G_{r_{L}}}{{{Re}_{L}}^{2}},\;{Re}_{L}=\frac{U_{\infty }L}{\nu },\;D_{al}=\frac{k}{{\epsilon^{+}L}^{2}},\;P_{r}=\frac{\nu }{\alpha },\;G_{r_{L}}=\frac{g\beta \Delta TL^{3}}{\nu^{2}}cos\alpha ,\;\gamma =\frac{\nu }{\nu_{m}},\;\Omega =\frac{1}{D_{al.{Re}_{L}}},\;\delta =\frac{Q_{o}L}{\rho C_{p}U_{\infty }}.$$ (13)

The stream velocity under |ε|<<1, where ε presents a small magnitude periodic component and the frequency parameter *ω* is takes the term *U*(*τ*) = 1+*ϵe*^*iωτ*^. The velocity of the fluid, magnetic field and temperature components *u*, *v*, *h*_*x*_, *h*_*y*_ and *θ* are defined as the sum of steady and unsteady equations.


$$\begin{array}{l} \;\bar{u}=u_{s}+\epsilon u_{t}e^{i\omega \tau },\;\bar{v}=v_{s}+\epsilon v_{t}e^{i\omega \tau },\;{\bar{h}}_{x}=h_{xs}+\epsilon h_{xt}e^{i\omega \tau }\\ {\bar{h}}_{y}=h_{ys}+\epsilon h_{yt}e^{i\omega \tau },\;\bar{\theta }=\theta_{s}+\epsilon \theta_{t}e^{i\omega \tau }. \end{array}$$ (14)

By following orders *O*(*ε*^0^) and *O*(*εe*^*iωτ*^), using these orders from Eqs (7–11) using proposed boundary-conditions (12), we can separately replace dimensionless steady and unsteady equations by using Eq (14) in the form of:

**For steady components:**

$$\frac{\partial u_{s}}{\partial x}+\frac{\partial v_{s}}{\partial y}=0$$ (15)

$$u_{s}\frac{\partial u_{s}}{\partial x}+v_{s}\frac{\partial u_{s}}{\partial y}=\frac{\partial^{2}u_{s}}{{\partial y}^{2}}+\xi \left(h_{xs}\frac{\partial h_{xs}}{\partial x}+h_{ys}\frac{\partial h_{xs}}{\partial y}\right)-\Omega u_{s}+\lambda \theta_{s}$$ (16)

$$\frac{\partial h_{xs}}{\partial x}+\frac{\partial h_{ys}}{\partial y}=0$$ (17)

$$u_{s}\frac{\partial h_{xs}}{\partial x}+v_{s}\frac{\partial h_{xs}}{\partial y}-h_{xs}\frac{\partial u_{s}}{\partial x}-h_{ys}\frac{\partial u_{s}}{\partial y}=\frac{1}{\gamma }\frac{\partial^{2}h_{s}}{\partial y^{2}}$$ (18)

$$u_{s}\frac{\partial \theta_{s}}{\partial x}+v_{s}\frac{\partial \theta_{s}}{\partial y}=\frac{1}{P_{r}}\frac{\partial^{2}\theta_{s}}{\partial y^{2}}\pm \delta \theta_{s}$$ (19)

with appropriate boundary conditions:

$$\begin{array}{l} \;u_{s}=v_{s}=0,\;h_{ys}=0,\;h_{xs}=1,\;\theta_{s}=1\;at\;y=0\\ u_{s}\to 1,\;\theta_{s}\to 0,\;h_{xs}\to 0\;asy\to \infty . \end{array}$$ (20)

By taking into consideration the Stokes second problem or sometimes referred to as oscillating boundary layer given in Eq (21) to split the unsteady variables into imaginary part and real part. Thus the separate form of the imaginary and real equations can be computed by using the equation Eq (21) given below.


$$\begin{array}{l} \;u_{t}=u_{1}+iu_{2},\;v_{t}=v_{1}+iv_{2},\;\theta_{t}=\theta_{1}+i\theta_{2},\\ h_{xt}=h_{x1}+ih_{x2},\;h_{yt}=h_{y1}+ih_{y2}. \end{array}$$ (21)

For real components:

$$\frac{\partial u_{1}}{\partial x}+\frac{\partial v_{1}}{\partial y}=0$$ (22)

$$-\omega u_{2}+u_{s}\frac{\partial u_{1}}{\partial x}+u_{1}\frac{\partial u_{s}}{\partial x}+v_{s}\frac{\partial u_{1}}{\partial y}+v_{1}\frac{\partial u_{s}}{\partial y}=\frac{\partial^{2}u_{1}}{{\partial y}^{2}}+\xi \left(h_{xs}\frac{\partial h_{x1}}{\partial x}+h_{x1}\frac{\partial h_{xs}}{\partial x}+h_{ys}\frac{\partial h_{x1}}{\partial y}+h_{y1}\frac{\partial h_{xs}}{\partial y}\right)-\Omega u_{1}+\lambda \theta_{1}$$ (23)

$$\frac{\partial h_{x1}}{\partial x}+\frac{\partial h_{y1}}{\partial y}=0$$ (24)

$$-\omega h_{x2}+u_{s}\frac{\partial h_{x1}}{\partial x}+u_{1}\frac{\partial h_{xs}}{\partial x}+v_{s}\frac{\partial h_{x1}}{\partial y}+v_{1}\frac{\partial h_{xs}}{\partial y}-h_{xs}\frac{\partial u_{1}}{\partial x}-h_{x1}\frac{\partial u_{s}}{\partial x}-h_{ys}\frac{\partial u_{1}}{\partial y}-h_{y1}\frac{\partial u_{s}}{\partial y}=\frac{1}{\gamma }\frac{\partial^{2}h_{x1}}{\partial y^{2}}$$ (25)

$$-\omega \theta_{2}+u_{s}\frac{\partial \theta_{1}}{\partial x}+u_{1}\frac{\partial \theta_{s}}{\partial x}+v_{s}\frac{\partial \theta_{1}}{\partial y}+v_{1}\frac{\partial \theta_{s}}{\partial y}=\frac{1}{P_{r}}\frac{\partial^{2}\theta_{1}}{\partial y^{2}}\pm \delta \theta_{1}$$ (26)

along with boundary conditions:

$$\begin{array}{l} \;u_{1}=v_{1}=0,\;h_{y1}=0,\;h_{x1}=0,\;\theta_{1}=0\;at\;y=0\\ u_{1}\to 1,\;\theta_{1}\to 0,\;h_{x1}\to 0\;as\;y\to \infty . \end{array}$$ (27)

**For imaginary components:**

$$\frac{\partial u_{2}}{\partial x}+\frac{\partial v_{2}}{\partial y}=0$$ (28)

$$\omega u_{1}+u_{s}\frac{\partial u_{2}}{\partial x}+u_{2}\frac{\partial u_{s}}{\partial x}+v_{s}\frac{\partial u_{2}}{\partial y}+v_{2}\frac{\partial u_{s}}{\partial y}=\frac{\partial^{2}u_{2}}{{\partial y}^{2}}+\xi \left(h_{xs}\frac{\partial h_{x2}}{\partial x}+h_{x2}\frac{\partial h_{xs}}{\partial x}+h_{ys}\frac{\partial h_{x2}}{\partial y}+h_{y2}\frac{\partial h_{xs}}{\partial y}\right)-\Omega u_{2}+\lambda \theta_{2}$$ (29)

$$\frac{\partial h_{x2}}{\partial x}+\frac{\partial h_{y2}}{\partial y}=0$$ (30)

$$\omega h_{x1}+u_{s}\frac{\partial h_{x2}}{\partial x}+u_{2}\frac{\partial h_{xs}}{\partial x}+v_{s}\frac{\partial h_{x2}}{\partial y}+v_{2}\frac{\partial h_{xs}}{\partial y}-h_{xs}\frac{\partial u_{2}}{\partial x}-h_{x2}\frac{\partial u_{s}}{\partial x}-h_{ys}\frac{\partial u_{2}}{\partial y}-h_{y2}\frac{\partial u_{s}}{\partial y}=\frac{1}{\gamma }\frac{\partial^{2}h_{x2}}{\partial y^{2}}$$ (31)

$$\omega \theta_{1}+u_{s}\frac{\partial \theta_{2}}{\partial x}+u_{2}\frac{\partial \theta_{s}}{\partial x}+v_{s}\frac{\partial \theta_{2}}{\partial y}+v_{2}\frac{\partial \theta_{s}}{\partial y}=\frac{1}{P_{r}}\frac{\partial^{2}\theta_{2}}{\partial y^{2}}\pm \delta \theta_{2}$$ (32)

along with boundary conditions:

$$\begin{array}{l} \;u_{2}=v_{2}=0,\;h_{y2}=0,\;h_{x2}=0,\;\theta_{2}=0,\;at\;y=0\\ u_{2}\to 0,\;\theta_{2}\to 0,\;h_{x2}\to 0,\;as\;y\to \infty . \end{array}$$ (33)

## 3. Solution methodology

The system of equations give in (15)–(19) and (22)–(32) along with boundary conditions have been transform into primitive form by following [28–30] for integration. Later, we use Finite Difference procedure which is based on the well-documented repute in literature. Spatial differencing schemes of second-order accuracy were used for the equation terms. A central differencing was selected for the diffusion terms (Ilyas et al. [29]) was adopted to discretize the nonlinear convective terms. The general form of the system of obtained algebraic equations can be expressed as.


$$A\Gamma_{(i-1,j)+}B\Gamma_{\left(i,j\right)}+C\Gamma_{\left(i+1,j\right)}=D$$

Here, Γ represent the field variable *u*, *v*, *θ*, *h*_*x*_ and *h*_*y*_ respectively, where *A*, *B*, *C* and *D* are coefficient matrices for above mentioned unknown variables. Later, the coefficient matrices are solved by using Gaussian elimination technique. Convergence of the solutions was declared at each step by following.


$$max|\Gamma^{n+1}-\Gamma^{n}|\leq 10^{-5}$$

Where *n* represent the nth iteration.

In actual computations, first the steady-state solutions are obtained and then are used for the solution of unsteady system of equations. Later, the obtained steady and unsteady solutions are used to calculate periodic skin friction *τ*_*s*_, heat transfer *τ*_*t*_ and current density *τ*_*m*_ along the electrically conducting cone, where *A*_*s*_, *A*_*t*_ and *A*_*m*_ are amplitudes while, *α*_*s*_, *α*_*t*_ and *α*_*m*_ are phase angles (see [30]).

$$\tau_{w}={\left(\frac{\partial U}{\partial Y}\right)}_{y=0}+\varepsilon |A_{s}|Cos(\omega t+\alpha_{s}),\;q_{w}={\left(\frac{\partial \theta }{\partial Y}\right)}_{y=0}+\varepsilon |A_{t}|Cos(\omega t+\alpha_{t}),$$ (34)

$$j_{w}={\left(\frac{\partial \varphi }{\partial Y}\right)}_{y=0}+\varepsilon |A_{m}|Cos(\omega t+\alpha_{m}),$$

where

$$A_{s}={\left({u_{1}}^{2}+{u_{2}}^{2}\right)}^{\frac{1}{2}},\;A_{t}={\left({\theta_{1}}^{2}+{\theta_{2}}^{2}\right)}^{\frac{1}{2}},\;A_{m}={\left({\varphi_{x1}}^{2}+{\varphi_{x2}}^{2}\right)}^{\frac{1}{2}},$$

αs=tan−1(u2u1),αt=tan−1(θ2θ1),αm=tan−1(φx2φx1).

## 4. Results and discussions for steadiness and periodical behavior

A model Stokes boundary layer problem in which buoyancy plays a significant role has been formulated for flow along a thermally and electrically conducting cone embedded in a porous medium. The situation of Stokes boundary layer depends on the effects of different parameters involved in the fluid flow model. The results also show that depending upon the orientation of the surface, buoyancy forces acting on the heated fluid near the surface of the cone alter the flow such that the processes is accelerated or delayed. The solutions for the various values of the parameters involved in the flow model show that there is a strong coupling between the fluid flow and heat transfer within the boundary layer. In this study, we are particularly concerned with the computational analysis of the characteristics of heat and fluid flow mechanism along the coordinate system depicted in Fig 1. With this understanding the detail discussion of the obtained results is given in the below paragraphs.

**Fig 2:**
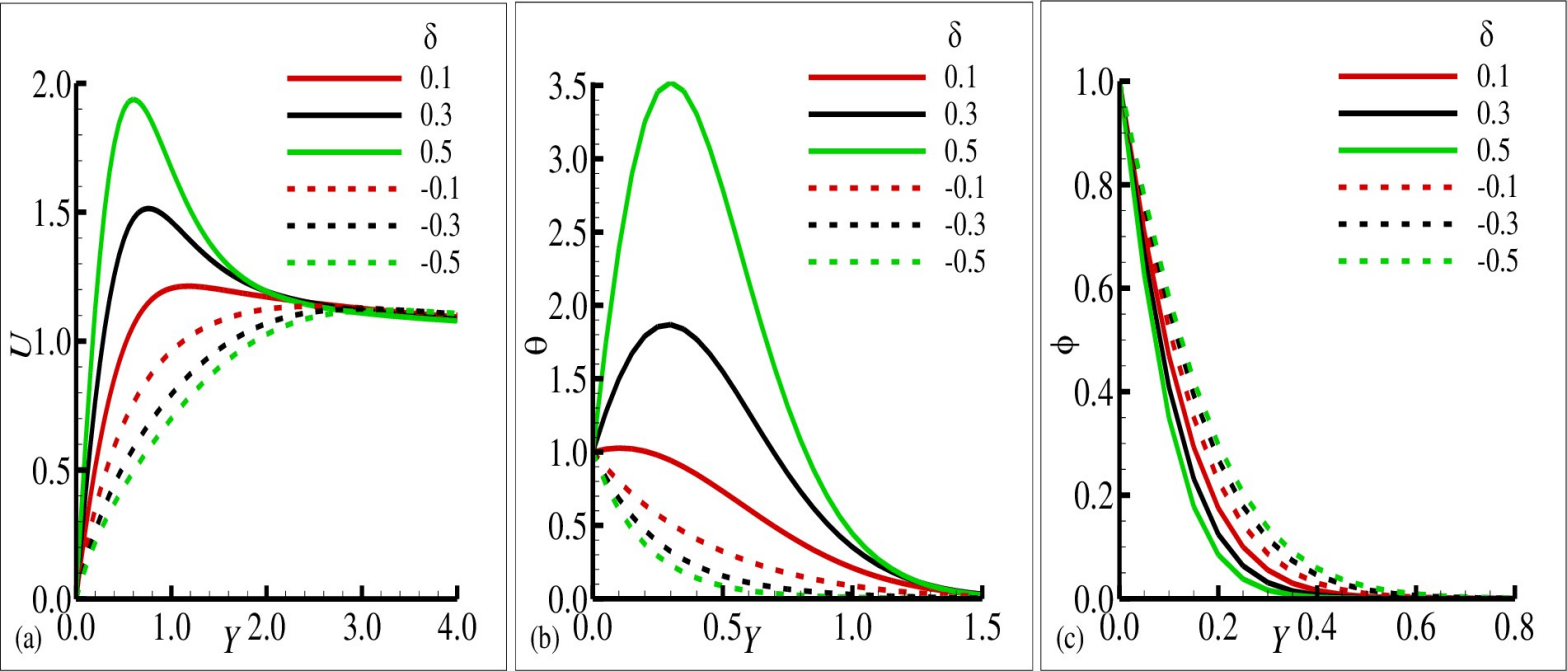
The graphical results for (a) velocity field *u*, (b) temperature field *θ*, and (c) magnetic field ϕ with various values of *δ* = 0.1, 0.5, 1.0 for heat source and *δ* = −0.1, −0.5, −1.0 for sink, where others are *γ* = 0.5, Pr = 7.0, *ξ* = 0.2, *λ* = 0.1 and Ω = 0.05.

**Fig 3:**
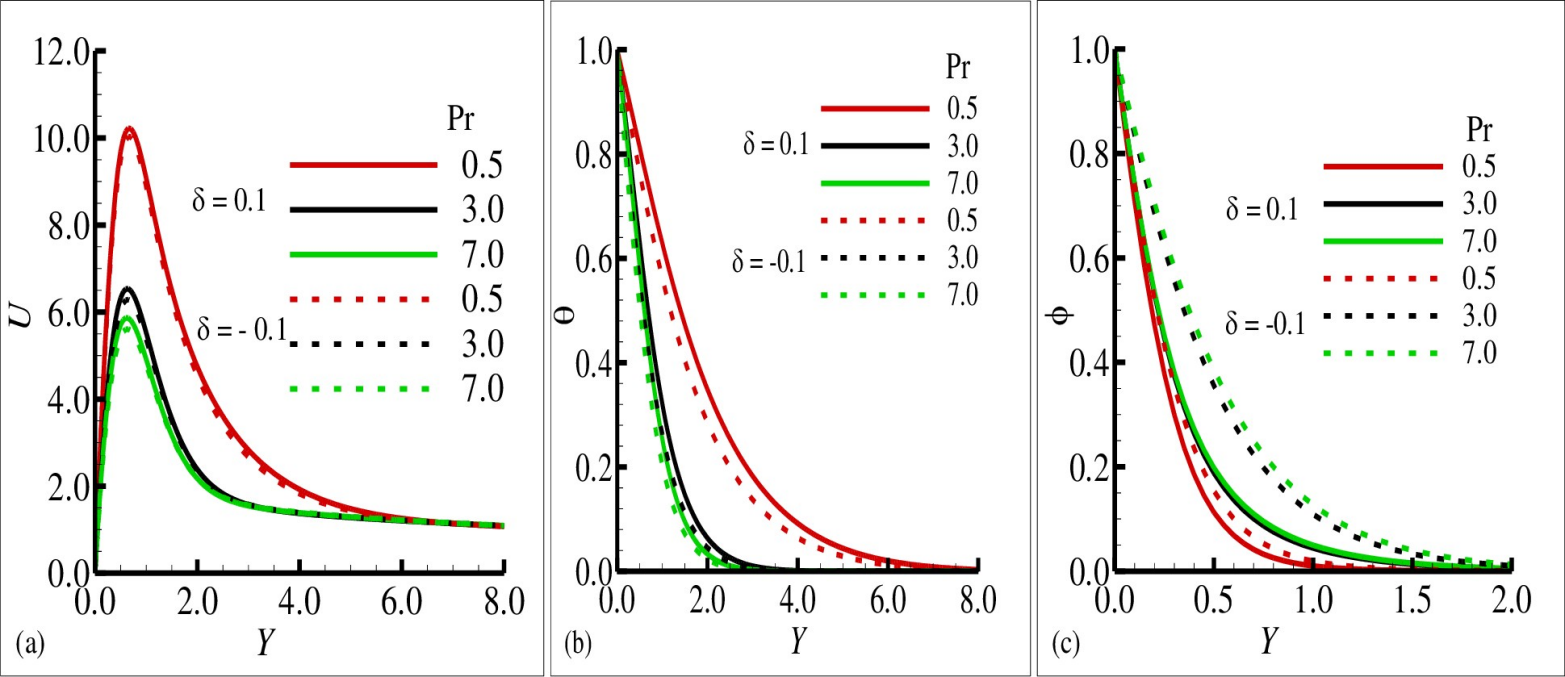
The graphical results for (a) velocity field *u*, (b) temperature field *θ*, and (c) magnetic field ϕ with choice values of Pr = 0.5, 3.0, 7.0 for heat source *δ* = 0.6 and sink *δ* = −0.6, where others are *γ* = 0.4, *λ* = 0.2, *ξ* = 0.8, and *Ω* = 0.5.

**Fig 4:**
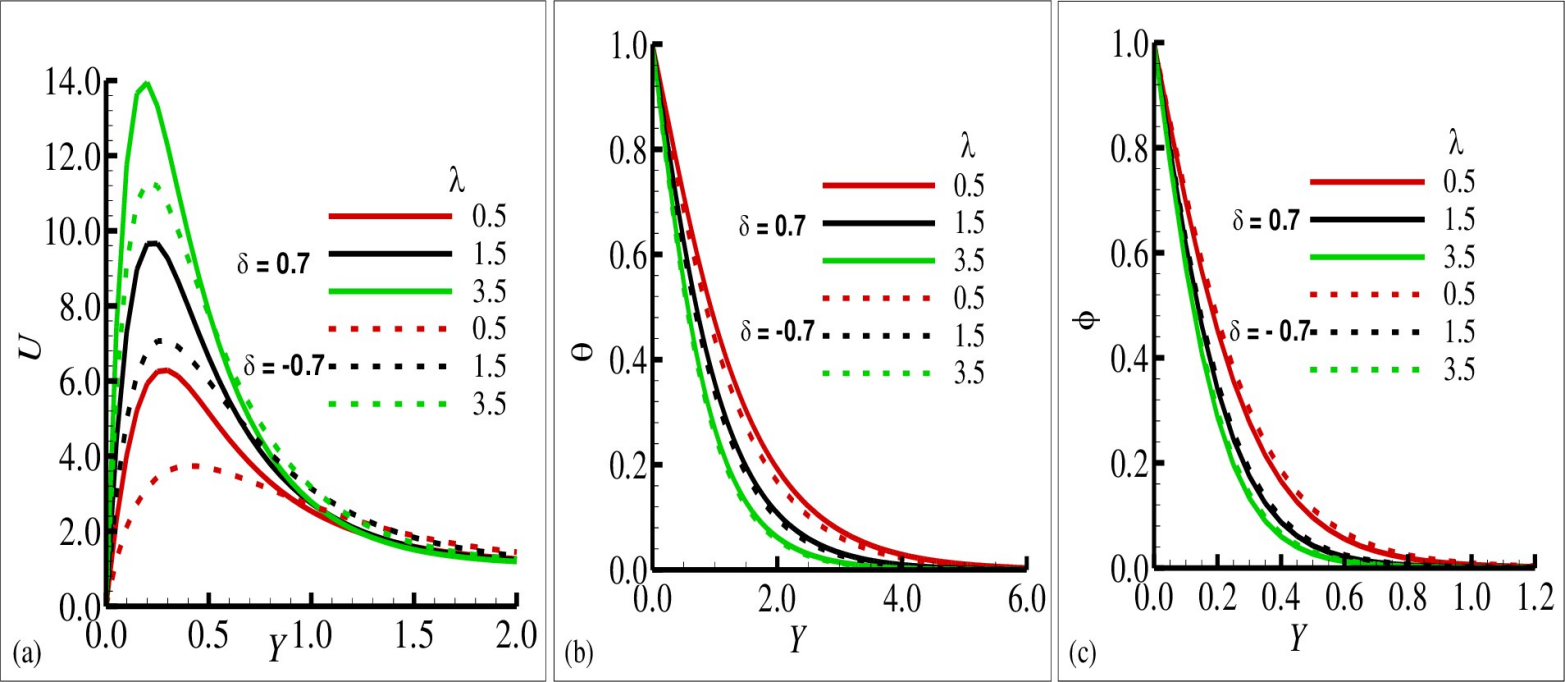
The graphical results for (a) velocity field *u*, (b) temperature field *θ*, and (c) magnetic field ϕ with the choice values of *λ* = 0.5, 1.5, 3.5 for heat source *δ* = 0.7 and sink *δ* = −0.7, where others are *γ* = 0.3, *Ω* = 0.06, *ξ* = 0.7 and Pr = 7.0.

**Fig 5:**
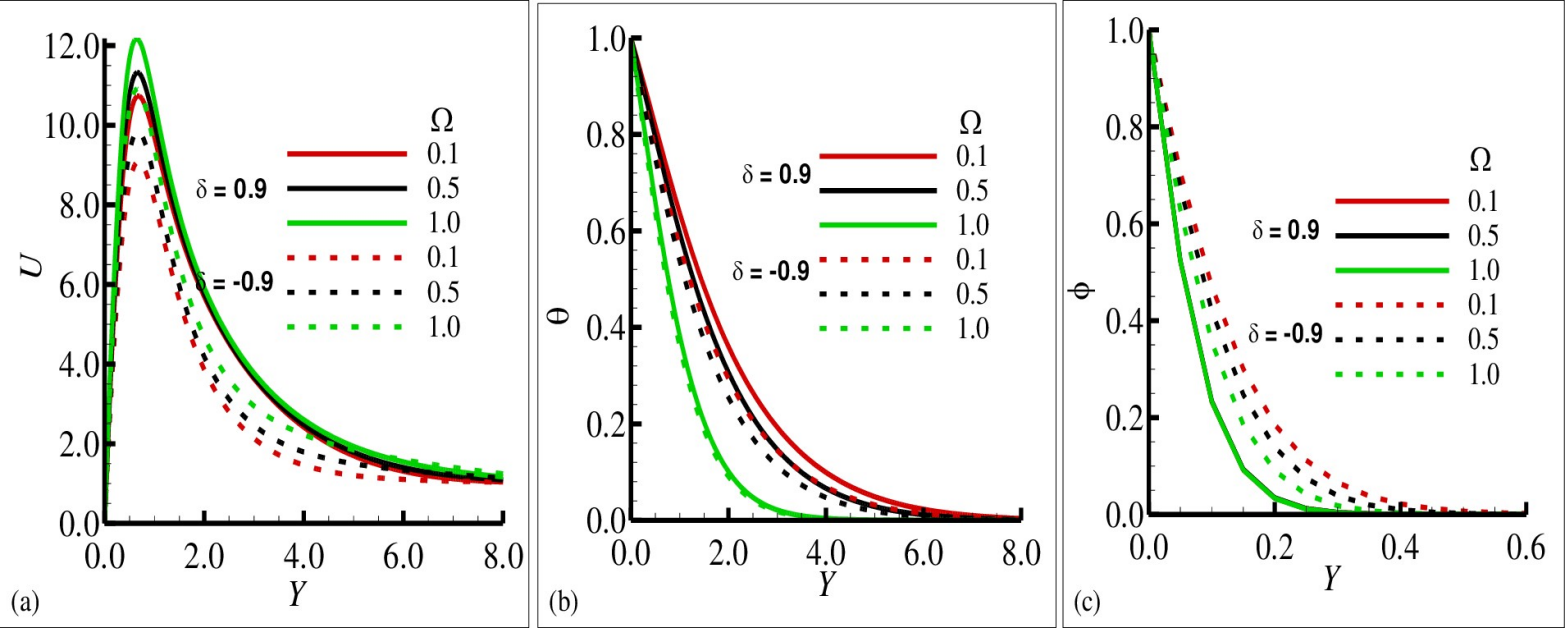
The graphical results for (a) velocity field *u*, (b) temperature field *θ*, and (c) magnetic field ϕ with the choice values of Ω = 0.1, 0.5, 1.0 for heat source *δ* = 0.9 and sink *δ* = −0.9, where others are *γ* = 0.4, Pr = 7.0, *ξ* = 0.5 and *λ* = 0.1.

Fig 2(A)–2(C) reported typical velocity, temperature, and transverse magnetic field profiles in the boundary layer along the surface of cone for various values of the source sink parameter ±*δ* ((+) sign indicates source and (–) sign indicates the sink), respectively. In these figures, it is shown that the velocity component *U* and the temperature variable (*θ*) is increased as the values of the source parameter (+*δ*) is increased and these components are reduced for the sink parameter (−*δ*). It is also shown the transverse component of magnetic field (*ϕ*) is reduced for the source parameter (+*δ*) but on the other hand it is slightly increased for the increasing values of sink parameter (−*δ*). It is pertinent to point out that the solid lines in each plot is seen the effects of source parameter while the dashed lines highlights the effects of sink parameter. The phenomena in Fig 3(A)–3(C) illustrate the effects of the various values of the Prandtl number Pr on the velocity component *U*, temperature variable (*θ*) and transverse magnetic field variable (*ϕ*) in the simultaneous presence of source and sink parameter (*δ* = ±0.6). It is concluded that the increase in Prandtl number Pr is reduced the velocity component *U* and the temperature variable (*θ*) in both cases that is source and sink while the transverse component of magnetic (*ϕ*) is increased in both source and sink regions. The effects of the mixed convection parameter *λ* on the velocity component, temperature variable and transverse magnetic field are reflected in Fig 4(A)–4(C), for source sink parameter *δ* = ±0.7 respectively. In these plots, it can be seen for increasing values of mixed convection parameter *λ* the velocity component *U* is increased and variable temperature and transverse magnetic field are reduced with the same trend simultaneously both for source and sink parameter (*δ* = ±0.7). Fig 5(A)–5(C) depict the influence of the porous medium parameter Ω on the velocity component *U*, temperature variable (*θ*) and transverse magnetic field (*ϕ*) for *δ* = ±0.9 respectively. It is observed that imposition of porous medium parameter increases the velocity component and decreases the temperature variable (*θ*) for source parameter and the transverse magnetic field is reduced for sink parameter (−*δ*) and no changes are observed for the case of source parameter (+*δ*).

**Fig 6:**
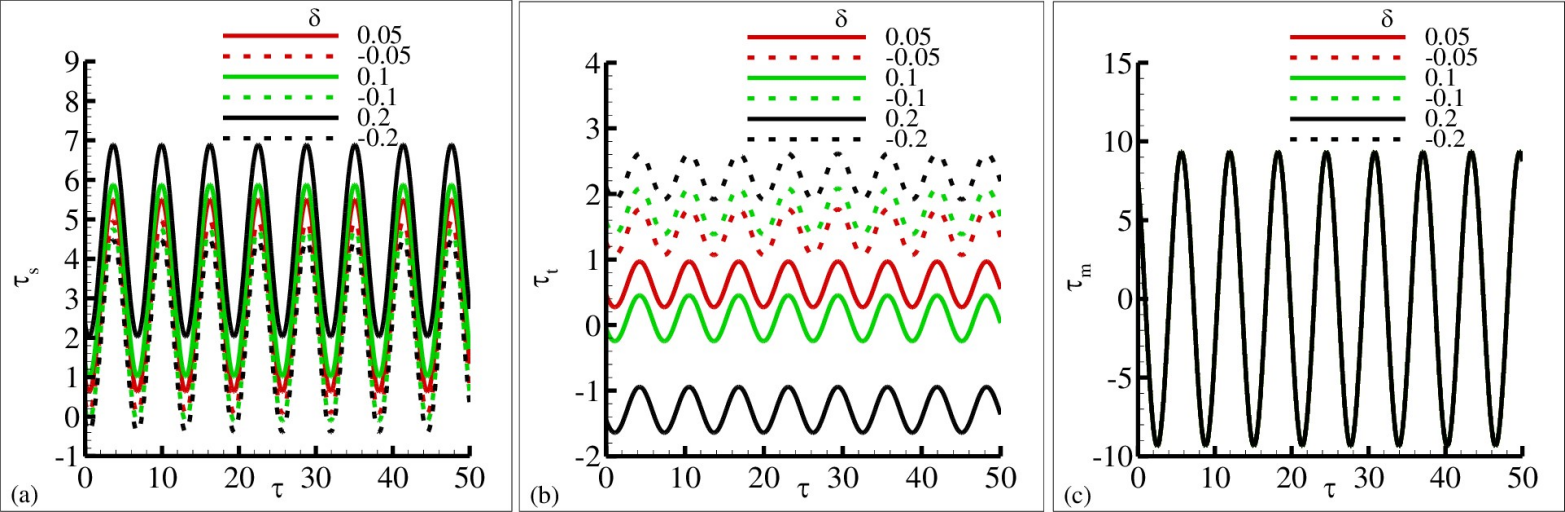
The graphical results of (a) *τ*_*w*_ (b) *q*_*w*_ and (c) *j*_*w*_ for various values of heat source *δ* = 0.05, 0.1, 0.2 and heat sink *δ* = −0.05, −0.1, −0.2, where other parameters *γ* = 1.6, *ξ* = 0.6, Pr = 7.0, Ω = 1.5 and *λ* = 7.5.

**Fig 7:**
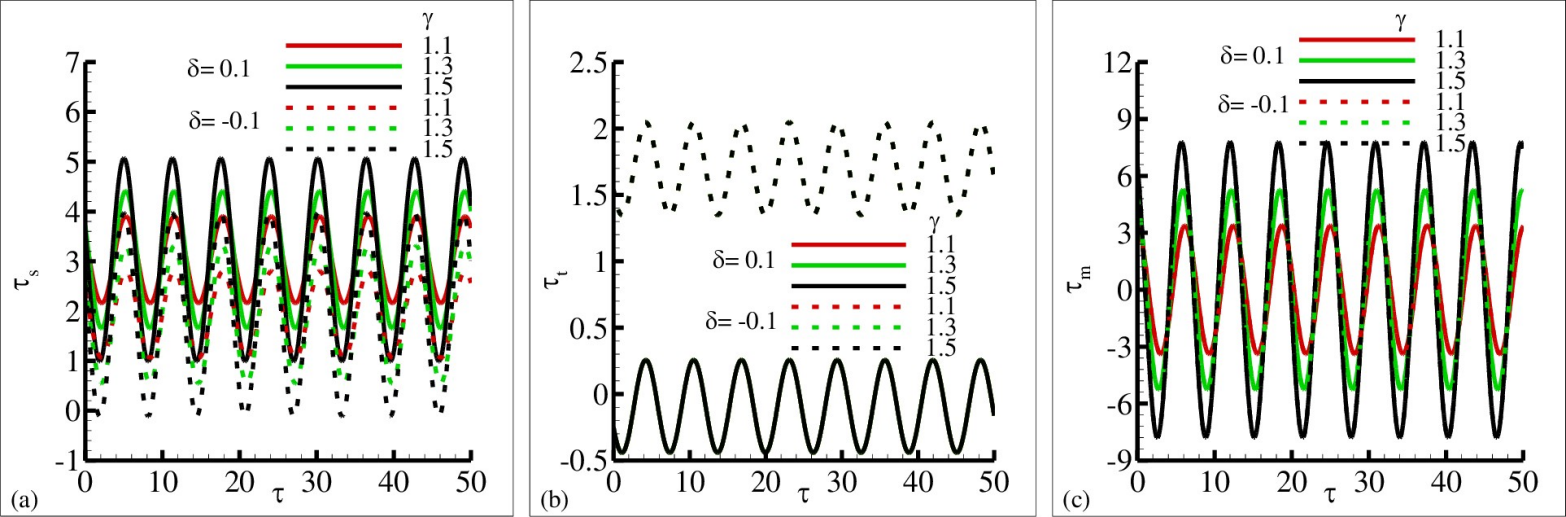
The graphical results of (a) *τ*_*w*_ (b) *q*_*w*_ and (c) *j*_*w*_ for various values of *γ* = 1.1, 1.3, 1.5 for heat source *δ* = 0.1 and sink *δ* = −0.1, where other parameters *λ* = 6.2, *ξ* = 0.2, Pr = 7.0, and *Ω* = 1.7.

**Fig 8:**
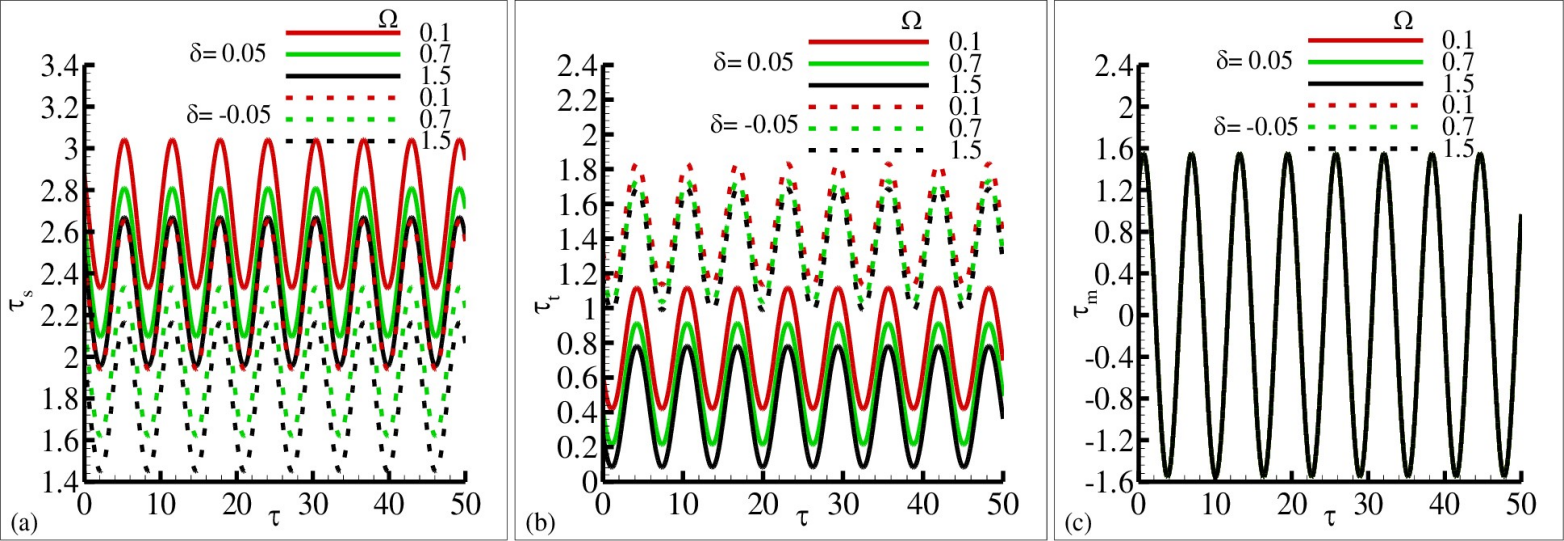
The graphical results of (a) *τ*_*w*_ (b) *q*_*w*_ and (c) *j*_*w*_ for various values of Ω = 0.1, 0.7, 1.5 for heat source *δ* = 0.05 and sink *δ* = −0.05, where other parameters *γ* = 0.8, *ξ* = 0.3, Pr = 7.0, and *λ* = 5.1.

**Fig 9:**
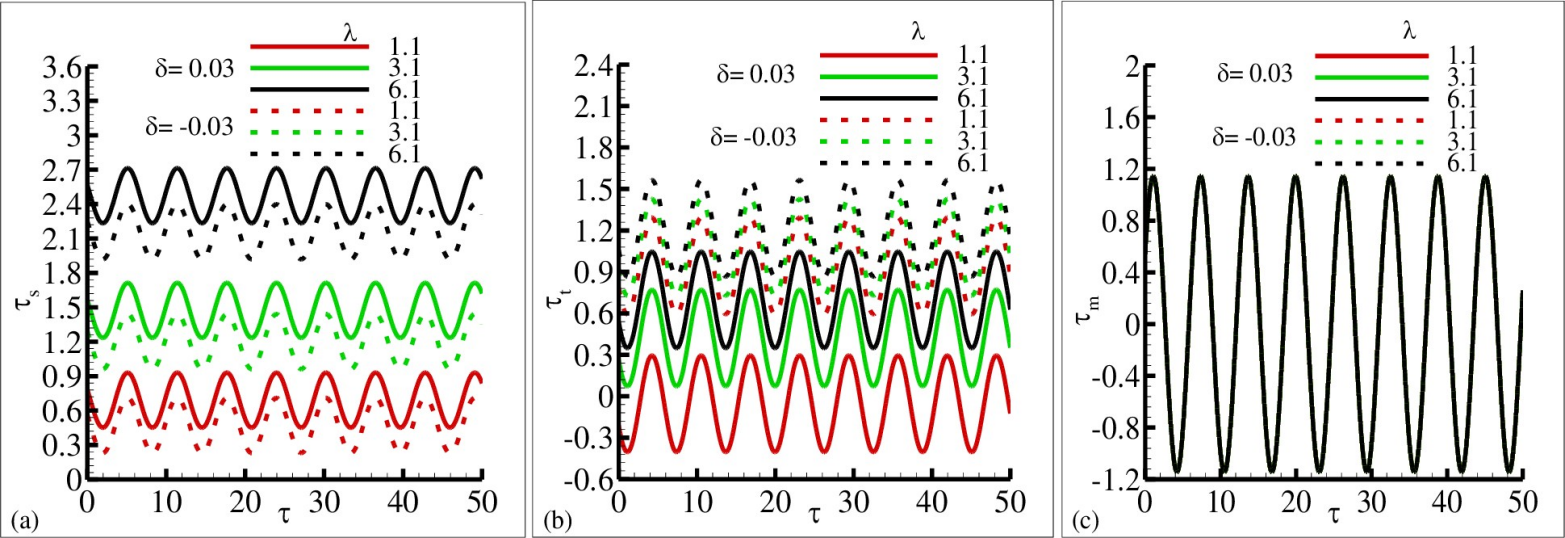
The graphical results of (a) *τ*_*w*_ (b) *q*_*w*_ and (c) *j*_*w*_ for various values of *λ* = 1.1, 3.1, 6.1 for heat source *δ* = 0.03 and sink *δ* = −0.03, where other parameters *Ω* = 1.7, *ξ* = 0.8, Pr = 7.0, and *γ* = 0.7.

The influence of the source and sink parameter ±*δ* on the periodical skin friction, heat transfer and current density is presented in Fig 6(A)–6(C), respectively. This is due to the reason that because of the increase in sink parameter the heated surface is transfer its energy to the fluid which is at lower temperature than the surface temperature. The second reason is that due to an increase in source parameter the constant heat parameter *Q*_*o*_ is increase and kinetic motion of the fluid particle is decreased. In these plots it is very clear that amplitude of periodic skin friction is increased for increasing values of source parameter *δ* and is reduced in the sink ranges. In the case of periodical heat transfer this trend is reversed, the amplitude of periodic heat transfer is increased in the sink range and reduced in the source range. It is important to point out that the periodical current density is uniform in both rages. The behavior of periodical skin friction, heat transfer and current density is highlighted in Fig 7(A)–7(C) for different values of magnetic Prandtl number *γ* in source and sink range respectively. From these figures it is depicted that the amplitude of periodic skin friction is increased for source range and is reduced for sink range, but the periodic heat transfer is increased in sink region and is uniformly distributed in each range. In Fig 7(C) it is noted that the periodic current density is increased with the increasing values of *γ* and particularly uniformly distributed in source and sink range. The physical reasoning depicted in Fig 7(A)–7(C) is due to the fact that for increasing values of magnetic Prandtl number *γ* the magnetic diffusion rate is dominant over viscous diffusion rate. In Fig 8(A)–8(C), the increase of porous medium parameter Ω clearly leads to significant decrease in both the periodic skin friction and heat transfer. However, there is no change is noted in the case of periodic current density. It was expected because for increasing values of the porous medium parameter Ω the total amount of void space accessible from the surface or phase boundary is increased. From the inspection of Fig 9(A)–9(C), it reveals that for increasing values of mixed convection parameter *λ* the amount of periodic skin friction is increased in the heat source range while the reverse trend is noted in the case of periodic heat transfer, mean to say the heat sink is significantly dominated. It is important to define that the mixed convection parameter is the ratio of the buoyancy force to flow shear force. The mechanism predicted in Fig 9(A)–9(C) is due to the reason for the increasing values of mixed convection parameter *λ* that buoyancy force is dominant over flow hear force.

## 5. Conclusions

In this paper, the occurrence of periodic/oscillatory convection flow along the surface of electrically conducting cone has been investigated numerically. For this purpose, comprehensive numerical solutions have been obtained to delineate the effect of different parameters involved in the flow model on velocity, temperature field and transverse magnetic field along with periodic skin friction, heat transfer and current density. Characteristics of the transient periodic/oscillatory convection appear largely in skin friction and heat transfer and in some cases of periodic current density. The main objective of this study is to investigate the impact of heat source and sink parameter periodic/oscillatory skin friction, heat transfer and current density.

In this study we conclude that intensity of heat and fluid flow is effective in the case of heat source and the heat sink effects are dominated by heat source. Moreover, heat source and sink are not prominent in the case of current density. It is noted that due to domination of the buoyancy force which acts like a pressure gradient the velocity profile, periodic skin friction is increased on the other hand temperature, transverse magnetic field, periodic heat transfer and current density are reduced. Due to the increase of porous medium parameter the avoid space accessible from the surface is increased thus a very significant reduction in periodic skin friction and heat transfer is noted. It is claimed that because of the prevailing attitude of the magnetic diffusion over viscous diffusion the velocity profile, temperature distribution, transverse magnetic field, periodic skin friction, periodic heat transfer, periodic current density are appreciably affected.

## Abbreviations

*T*_∞_ (K): Ambient-temperature
*u*, *v* (m s ^-1^): Velocity along *xy*-direction
$R_{e_{L}}$: Reynolds number
*H*_*x*_, *H*_*y*_: Magnetic field along *xy*-direction (Tesla)
$G_{r_{L}}$: Grashof number
*ν* (m^2^ s^-1^): Kinematic viscosity
*C*_*p*_ (J kg^-1^ K^-1^): The specific-heat
*σ* (s m^-1^): Electrical conductivity
*μ* (kg m^-1^ s^-1^): Dynamic viscosity
*τ* (P a): Shearing stress
*ρ* (kg m^-3^): Density
ξ: Magnetic−force number
*g* (m s^-2^): Gravitational-acceleration
λ: Mixed-convection number
*β* (K^-1^): Thermal-expansion
θ: Dimensionlized Temperature
*ν*_*m*_ (H m^-1^): Magnetic-permeability
γ: Magnetic-Prandtl parameter
*α* (m^2^ s^-1^): Thermal-diffusivity
Pr: Prandtl number
T (K): Fluid Temperature
